# Integrated geophysical, mineralogical, and geochemical investigation of gold and associated hydrothermal minerals in the Um Balad Area, Eastern Desert, Egypt

**DOI:** 10.1038/s41598-026-54382-7

**Published:** 2026-06-04

**Authors:** Mohamed H.Mahmoud, Mahmoud Abd El-Rahman Hegab, Abdelhamid Elbshbeshi, Sultan A. S. Araffa

**Affiliations:** 1 National Research Institute of Astronomy and Geophysics (NRIAG), Helwan, Cairo, 11421 Egypt; 2 Department of Earth and Environmental Sciences, Rutgers, The State University of New Jersey, Jersey City, 07102 USA; 3 Mineral Resources Department, National Authority for Remote Sensing and Space Sciences, Cairo, Egypt

**Keywords:** Gold, Gravity derivatives, Um Balad, ASD spectrometer, Scanning Electron Microscope (SEM), Environmental sciences, Solid Earth sciences

## Abstract

Gold exploration in structurally complex Precambrian terranes remains challenging due to the heterogeneous distribution of hydrothermal alteration zones and the difficulty of distinguishing mineralization-related structures from regional tectonic features. Recent advances in integrated geophysical interpretation have demonstrated the importance of combining multiple datasets to improve structural targeting and hydrothermal mapping within the Arabian–Nubian Shield. The Um Balad area is a promising target for gold and copper exploration, with widespread hydrothermal alteration associated with quartz veins and sulfide mineralization. This study integrates airborne magnetic data, ground gravity surveys, geochemical analyses, and mineralogical investigations to characterize the structural framework and hydrothermal alteration related to mineralization in the area. Gravity and magnetic datasets were processed to delineate lithological boundaries, structural discontinuities, and intrusive bodies associated with hydrothermal activity. Structural interpretation reveals dominant NW–SE, NNW–SSE, E–W, NE–SW, and NNE–SSW fault systems controlling fluid migration and alteration zones. Processing techniques, including radially averaged power spectrum analysis, single-body modeling, and Euler deconvolution were applied to estimate the depth extent of magnetic and gravity sources, indicating both shallow and deep-seated causative bodies. Geochemical and mineralogical analyses were conducted on representative samples from the metagabbro–diorite complex and older granitoids using Spectral Analysis (ASD) and SEM–EDX (Scanning Electron Microscopy combined with Energy Dispersive X-ray Spectroscopy). The identified alteration assemblages include hematite, goethite, muscovite, chlorite, illite, kaolinite, and ferrihydrite. SEM–EDX results indicate significant enrichment in Au, Cu, and Ag, with maximum concentrations of 5.69 wt.% Au, 1.49 wt.% Cu, and 0.22 wt.% Ag. The integration of geophysical, mineralogical, and geochemical datasets demonstrates that structurally controlled hydrothermal alteration zones represent the principal targets for gold mineralization in the Um Balad area.

## Introduction

Hydrothermal gold mineralization within Precambrian crystalline terranes is commonly controlled by structurally focused fluid circulation associated with shear zones, fault intersections, intrusive contacts, and deformation corridors. Identifying these structurally controlled mineralization pathways remains one of the principal challenges in mineral exploration, particularly within highly deformed basement complexes where lithological heterogeneity and multi-phase tectonic overprinting obscure subsurface relationships. Consequently, integrated geophysical approaches have become increasingly important for resolving structural architecture and mapping hydrothermal alteration systems associated with ore deposition.

The Um Balad area is situated in the northeastern sector of the Eastern Desert in Egypt along the Red Sea coast, northwest of Hurghada, between latitudes 27°48′00″–27°52′42″ N and longitudes 32°42′22″–32°52′20″ E. The area forms part of the Arabian–Nubian Shield (ANS), which represents one of the most important Neoproterozoic metallogenic provinces, hosting numerous gold occurrences associated with shear zones, quartz veins, and hydrothermal alteration systems^1,2^. Gold mineralization in the Eastern Desert is mainly hosted within metavolcanics, ophiolitic assemblages, granitoids, and gabbro–diorite complexes. The mineralization is structurally controlled and commonly associated with fault systems, shear zones, and quartz–carbonate veins formed during late-stage tectonic deformation and hydrothermal fluid circulation^3–8^.

The Um Balad area lies within this metallogenic framework and contains metagabbro–diorite complexes, Dokhan volcanics, Hammamat sediments, and granitoids^9,10^. The gold in the region is mainly associated with quartz veins and hydrothermal alteration zones enriched in iron oxides and clay minerals^11^. Hydrothermal alteration minerals such as hematite, chlorite, illite, kaolinite, muscovite, and goethite are important indicators of fluid–rock interaction and mineralization processes^5,12^. These minerals display distinctive spectral signatures that allow their identification through remote sensing and field spectrometry methods^13,14^.

Integrated geophysical techniques are widely applied in mineral exploration because they provide information about lithological contacts, structural trends, intrusive bodies, and alteration zones. Aeromagnetic data are effective for mapping magnetic susceptibility contrasts and structural lineaments, whereas gravity data are useful for detecting density contrasts associated with intrusive rocks, faults, and hydrothermal systems^15^. Derivative-based filtering techniques and depth estimation methods, including tilt derivative, power spectrum analysis, and Euler deconvolution, enhance structural interpretation and estimate the geometry of subsurface sources^16–19^.

Previous investigations in the Um Balad area mainly focused on remote sensing and gamma-ray spectrometry for alteration mapping^20–30^. However, the integration of airborne magnetic data, gravity surveys, spectral measurements, and SEM–EDX mineralogical analyses for understanding the structural controls and hydrothermal alteration associated with gold mineralization remains limited. Therefore, the present study aims to:

- Delineate the structural framework controlling mineralization using airborne magnetic and gravity data.
- Estimate the depth and geometry of magnetic and gravity sources using spectral analysis, Euler deconvolution, and forward modeling.
- Characterize hydrothermal alteration minerals using ASD spectrometry and SEM–EDX analysis.
- Integrate geophysical, mineralogical, and geochemical datasets to identify prospective exploration targets in the Um Balad area.

Despite extensive mineral exploration within the Arabian–Nubian Shield, several studies remain largely dependent on conventional interpretation approaches using isolated magnetic filtering techniques without quantitative integration of multi-source datasets. In the Um Balad area, previous investigations have mainly focused on geological mapping, remote sensing, and localized geochemical analyses, while integrated structural interpretation combining magnetic, gravity, mineralogical, and geochemical datasets remains limited. Furthermore, few studies have attempted to evaluate the spatial relationship between structural complexity, hydrothermal alteration mineralogy, and elemental enrichment patterns using a unified exploration framework. This limits the understanding of the structural controls governing hydrothermal fluid migration and mineral deposition.

### Scientific contribution and methodological significance

Although individual processing techniques such as RTP transformation, Euler deconvolution, and spectral analysis are well established, the novelty of the present study lies in the integrated multi-source interpretation framework rather than the isolated application of individual filters. The study combines:

- multi-scale magnetic and gravity structural enhancement,
- CET structural complexity analysis,
- spectral depth estimation,
- ASD mineral mapping,
- and SEM–EDX geochemical characterization

within a single structurally constrained exploration workflow. The integration strategy reduces interpretational ambiguity by linking geophysical discontinuities with independently verified mineralogical and geochemical indicators of hydrothermal activity. This process-based integration improves structural targeting in highly deformed Precambrian terranes and provides an adaptable framework for mineral exploration in analogous crystalline basement provinces.

### Geological setting

#### Regional geology

The Um Balad area is located in the northern Eastern Desert of Egypt, within the Arabian–Nubian Shield (ANS), which formed during the Neoproterozoic Pan-African orogeny. Metavolcanic sequences, ophiolitic assemblages, granitoids, Dokhan volcanics, Hammamat sediments, and post-tectonic granites characterize the regional geology. These rock units were affected by multiple tectonic deformation events associated with accretion, collision, shearing, and magmatism during the evolution of the ANS^1–3^.

The Eastern Desert is dissected by major NW–SE, NNW–SSE, and NE–SW fault systems that played an important role in magma emplacement and hydrothermal fluid migration. Gold mineralization in the region is commonly associated with structurally controlled quartz veins and shear zones developed within metavolcanics and intrusive complexes^2,4^.

#### Local geology

**Fig. 1:**
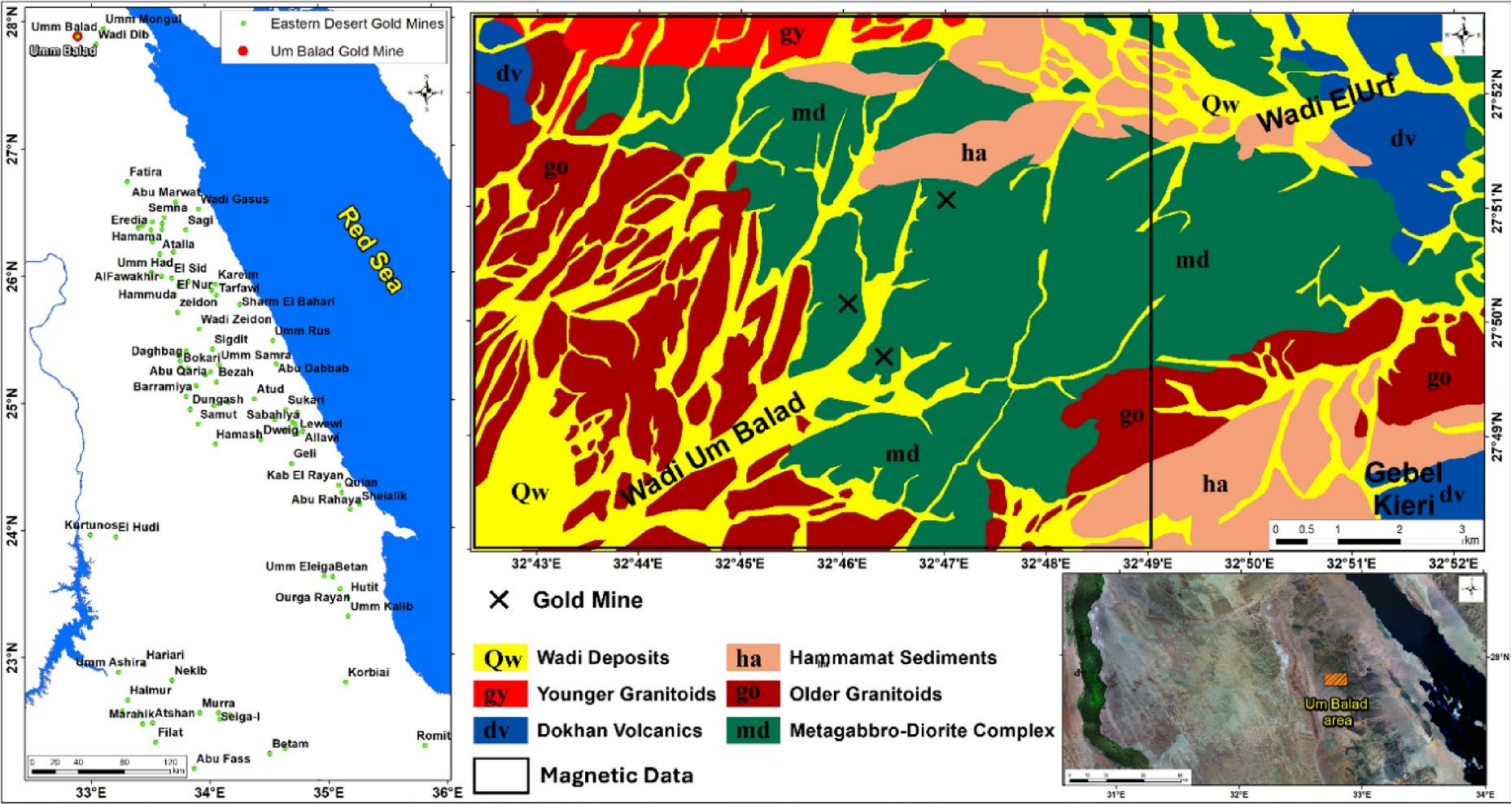
Maps showing the Um Balad area in Egypt’s North Eastern Desert, as well as the geology of the study area, and the locations of the country’s active gold mines.

The Um Balad area consists mainly of metagabbro–diorite complexes, older granitoids, Dokhan volcanics, and Hammamat sedimentary rocks^9,10^. The metagabbro–diorite rocks are highly fractured and altered, particularly along shear zones and fault intersections. Gold mineralization mainly occurs within quartz–carbonate veins associated with hydrothermal alteration zones rich in iron oxides and clay minerals^11^ (Fig. 1).

Hydrothermal alteration in the study area includes hematization, chloritization, silicification, kaolinization, and argillic alteration. NW–SE represents structural deformation and NE–SW trending faults and shear zones that control the distribution of quartz veins and mineralized zones. These structures acted as conduits for hydrothermal fluids and played a major role in the localization of mineralization.

## Materials and methods

### Data acquisition and materials

An integrated geophysical, mineralogical, and geochemical approach was adopted in this study to investigate the structural framework and hydrothermal alteration associated with gold mineralization in the Um Balad area of the northern Eastern Desert, Egypt. The methodology combined airborne magnetic data, ground gravity measurements, ASD spectrometry, and scanning electron microscopy coupled with energy-dispersive X-ray spectroscopy (SEM–EDX).

### Airborne magnetic data

The airborne magnetic data used in this study were obtained from the airborne geophysical surveys conducted by the Egyptian Geological Survey and Mining Authority (EGSMA). The survey covered the Um Balad region using a fixed-wing aircraft equipped with a high-sensitivity cesium vapor magnetometer. The survey specifications included: Flight-line spacing: approximately 500 m, Tie-line spacing: approximately 5 km, Mean terrain clearance: approximately 120 m, Flight direction: NE–SW, and Tie-line orientation: NW–SE.

The magnetic data were recorded in nanotesla (nT) and referenced to the World Geodetic System (WGS84). The International Geomagnetic Reference Field (IGRF) correction was previously applied to remove the regional geomagnetic field component.

### Ground gravity data

Ground gravity measurements were conducted to delineate subsurface density variations associated with lithological contacts, intrusive bodies, and fault systems related to mineralization. Gravity stations were distributed along accessible traverses covering the main geological units and structural trends of the study area.

A Scintrex CG-6 gravimeter was used for the gravity survey. Differential GPS measurements were employed to determine station elevations and coordinates with high positional accuracy. The ground gravity survey represents one of the strengths of the present study. A total of 219 gravity stations were acquired over a relatively limited area, with station spacing ranging from approximately 50 to 100 m depending on topographic accessibility and geological complexity. This relatively dense acquisition geometry improved the resolution of near-surface density contrasts and enhanced the delineation of structurally controlled anomalies associated with intrusive bodies and hydrothermal systems.

### Rock samples and laboratory analyses

Representative rock samples were collected from mineralized quartz veins, altered metagabbro–diorite rocks, and granitoids within structurally controlled alteration zones. The samples were subjected to mineralogical and geochemical analyses to characterize hydrothermal alteration minerals and ore-related elements.

#### ASD spectral measurements

Spectral measurements were performed using an ASD TerraSpec spectrometer over the wavelength range of 350–2500 nm. The spectral data were analyzed using reference spectral libraries to identify diagnostic absorption features of hydrothermal alteration minerals, including hematite, chlorite, muscovite, kaolinite, illite, and goethite.

#### SEM–EDX analysis

Selected polished samples were analyzed using scanning electron microscopy coupled with energy-dispersive X-ray spectroscopy (SEM–EDX) to determine mineral composition and elemental concentrations. Prior to analysis, the samples were polished and carbon-coated to improve conductivity. Quantitative elemental analysis was performed using ZAF correction procedures.

### Aeromagnetic data processing

**Fig. 2:**
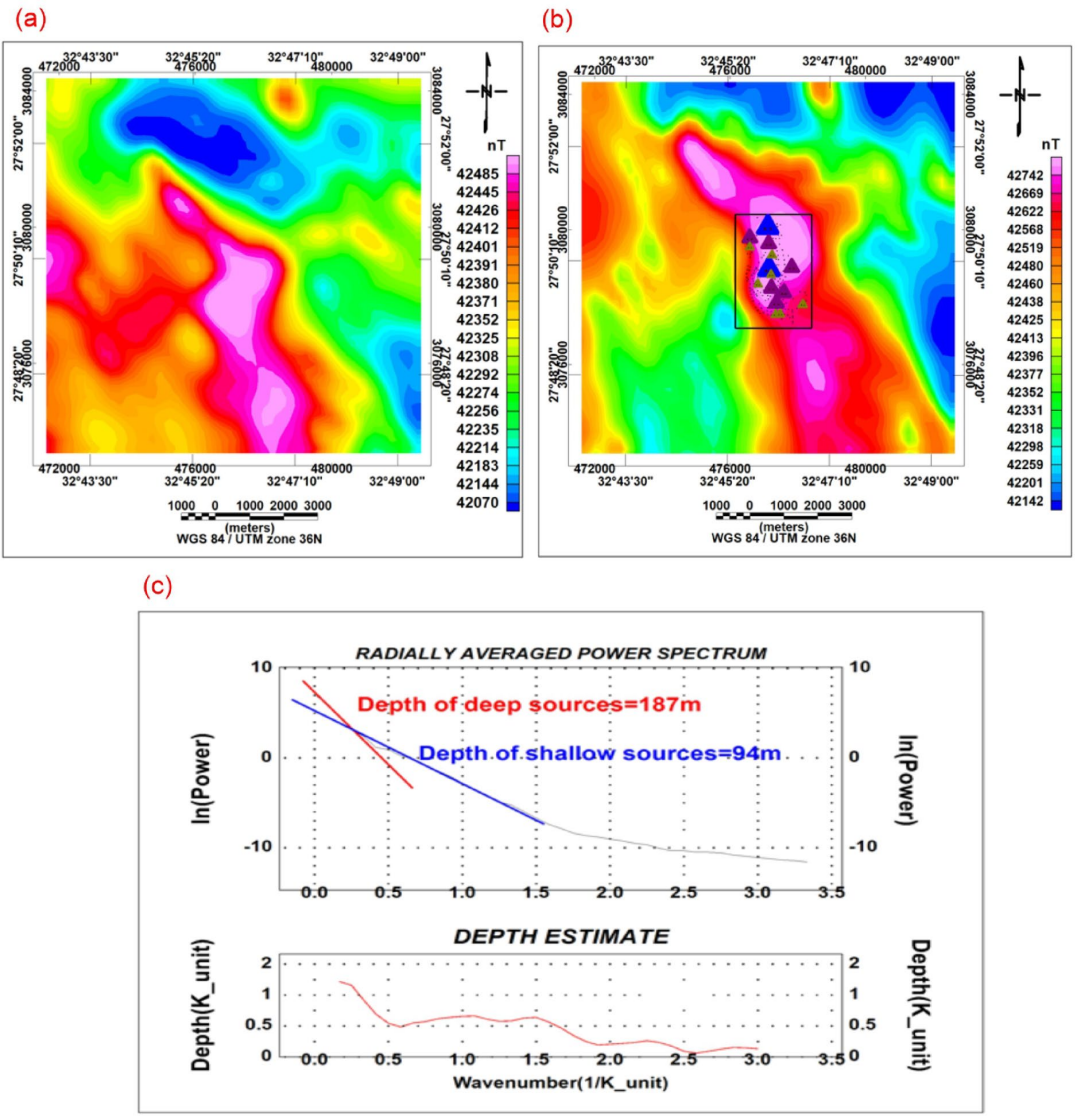
(**a**) Total magnetic intensity (TMI) map, (**b**) Reduced-to-Pole (RTP) magnetic anomaly map, (**c**) Radially averaged power spectrum of magnetic data.

The airborne magnetic dataset was processed using several filtering and enhancement techniques to delineate subsurface structures, lithological contacts, and magnetic source distributions associated with hydrothermal alteration and mineralization. Data processing and interpretation were carried out using Oasis montaj^31^ and related geophysical interpretation modules and the results were represented as a total magnetic intensity map (TMI) (Fig. 2a).

### Reduction to the pole (RTP)

Reduction to the Pole (RTP) transformation was applied to reposition magnetic anomalies directly above their causative sources. RTP processing minimizes the asymmetrical distortion of magnetic anomalies caused by the inclination and declination of the Earth’s magnetic field, particularly in low magnetic latitude regions such as Egypt. The RTP transformation enhances the interpretability of magnetic anomalies and improves the delineation of geological contacts and intrusive bodies. Due to Egypt’s low magnetic latitude, anomalies were asymmetrical and displaced from their causative sources. Therefore, reduction to the Pole (RTP) (Fig. 2b) was applied to transform the magnetic anomalies as if they were measured at the magnetic pole^15^.

In the frequency domain, the RTP operator is given by:

1 $$RTP({k}_{x},{k}_{y})=\frac{\left(i{k}_{x}+i{k}_{y}\mathrm{t}\mathrm{a}\mathrm{n}I\mathrm{s}\mathrm{i}\mathrm{n}D{)}^{2}\right.}{{k}^{2}}$$

where:$I$ =  = magnetic inclination, $D$= magnetic declination,$k=\sqrt{{k}_{x}^{2}+{k}_{y}^{2}}$.

The resulting RTP map centers anomalies over causative bodies and facilitates structural interpretation. Uncertainty associated with RTP processing arises from inaccuracies in inclination and declination parameters. Sensitivity testing (± 2° variation) resulted in anomaly shifts of < 5%, within acceptable interpretational limits.

### Regional–residual separation for magnetic data

**Fig. 3:**
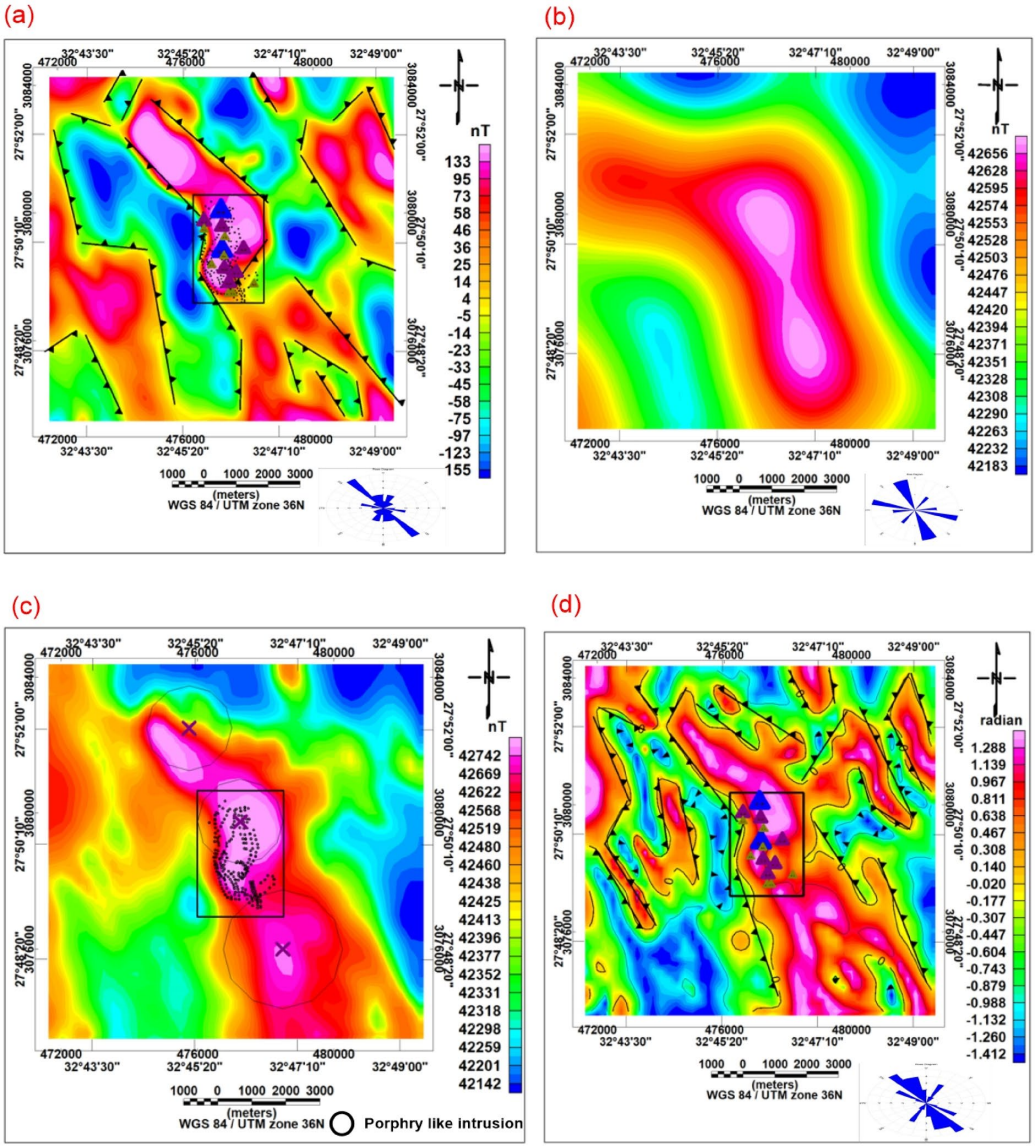
(**a**) Magnetic residual anomaly (high-pass filter), (**b**) Magnetic regional anomaly (low-pass filter), (**c**) CET porphyry feature extraction map, (**d**) Tilt derivative map of magnetic data.

Regional and residual magnetic components were separated to distinguish deep-seated geological structures from shallow subsurface features. Frequency-domain filtering techniques were applied using appropriate cutoff wavelengths of 0.55494 1/k_unit selected based on the spectral characteristics of the magnetic field^17^ (Fig. 2c). The regional component represents broad, deep-crustal features (Fig. 3a), whereas the residual component highlights shallow geological structures related to faults, dykes, alteration zones, and mineralized bodies (Fig. 3b).

### Derivative and edge enhancement techniques

Several derivative-based filters were applied to enhance structural discontinuities and lithological boundaries.

### Tilt derivative (TDR) for magnetic data

The tilt derivative was applied to improve the detection of subtle structural boundaries and weak magnetic contrasts. The TDR method is effective for mapping fault systems and contact zones, independent of variations in anomaly amplitude. The structural features are delineated tilt derivative technique (Fig. 3d). The tilt derivative is expressed as^32^:

2 $$TDR={\mathrm{t}\mathrm{a}\mathrm{n}}^{-1}\left(\frac{\partial T/\partial z}{\sqrt{\left(\partial T/\partial x{)}^{2}+(\partial T/\partial y{)}^{2}\right.}}\right)$$

Zero-contour lines of TDR correspond to source edges and fault contacts.

### CET structural analysis of magnetic data

The Centre for Exploration Targeting (CET) grid analysis techniques were applied to identify structural complexity and hydrothermal alteration patterns. CET analysis included texture analysis, phase symmetry, and lineament extraction algorithms. These processes assist in identifying structurally controlled mineralized zones by enhancing fault intersections, alteration corridors, and lineament density patterns. To demonstrate these characteristics and related hydrothermal alteration, the RTP magnetic data were analyzed using the CET-porphyry approach (Fig. 3c). The intrusion and its associated alteration zone exhibit higher magnetic values than the surrounding unaltered areas^20^.

### Gravity data processing

Gravity data processing was conducted to identify subsurface density contrasts associated with intrusive rocks, structural discontinuities, and hydrothermal systems.

### Gravity corrections

Several standard corrections were applied to the raw gravity measurements to obtain Bouguer gravity anomalies, including: Instrument drift correction, Latitude correction, Free-air correction, Bouguer correction, and Terrain correction^30^.

A standard rock density value of 2.67 g/cm^3^ was used for Bouguer correction calculations.

### Residual gravity analysis

Regional gravity trends were separated from residual anomalies using polynomial fitting and wavelength filtering techniques. Residual gravity anomalies were used to identify shallow density variations associated with fault zones and intrusive bodies (Fig. 5c).

### Gravity enhancement techniques

**Fig. 4:**
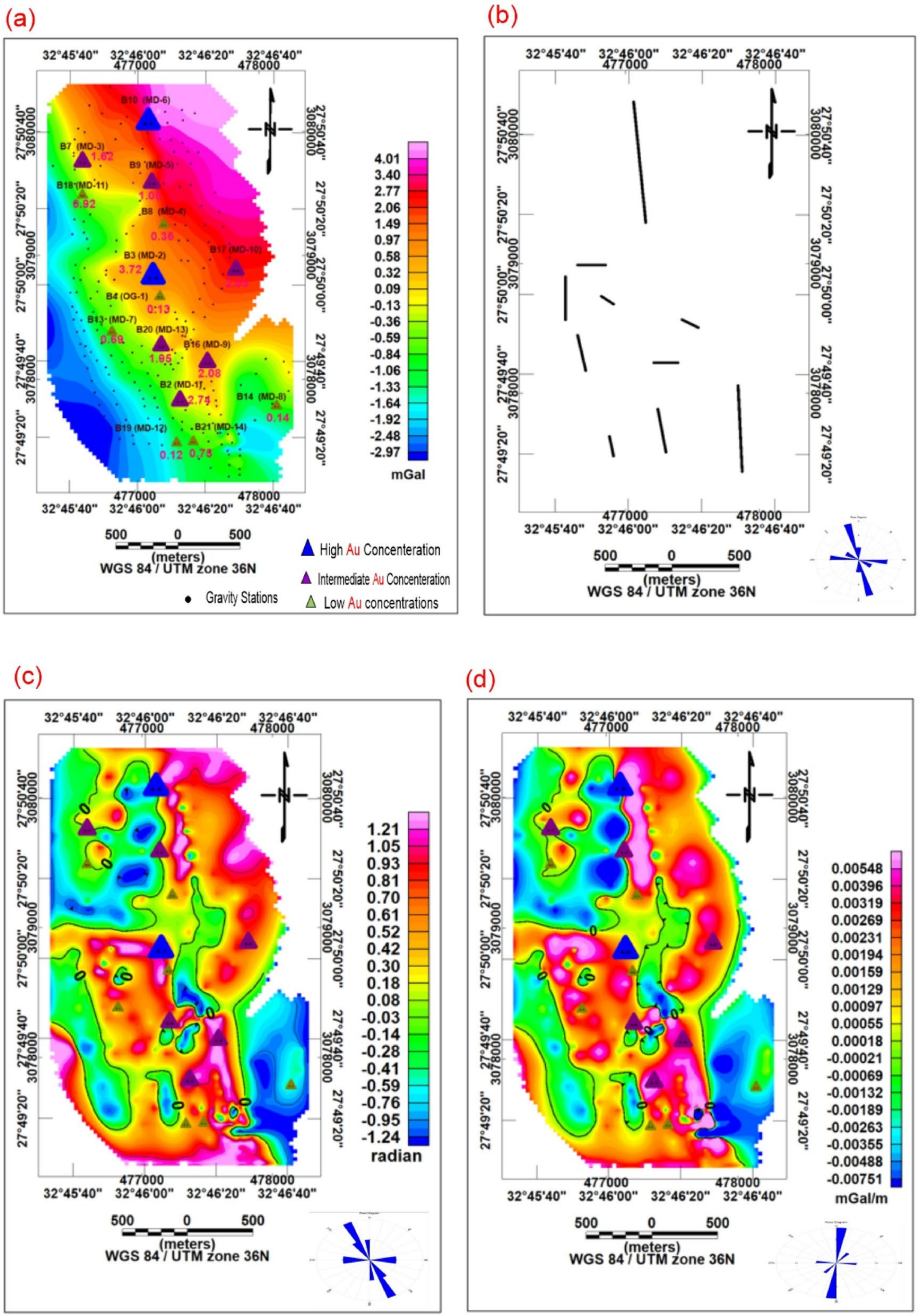
(**a**) Bouguer gravity anomaly map, (**b**) CET structural map derived from Bouguer data, (**c**) Tilt derivative of Bouguer anomaly, (**d**) First vertical derivative (FVD) of Bouguer anomaly.

Several enhancement filters were applied to improve the structural interpretation of the gravity data, including the Tilt derivative (Fig. 4c) and First vertical derivative (Fig. 4d)^15^. These filters improve the delineation of lithological boundaries and fault systems controlling hydrothermal fluid migration^31^.

### CET gravity structural analysis

CET grid analysis was also applied to gravity data to extract structural trends and identify areas of enhanced structural complexity associated with mineralization pathways^21^ (Fig. 4b). Gold and base-metal deposits commonly develop along significant structural features, such as faults, shear zones, dykes, and rock contacts, which serve as conduits for hydrothermal fluids. Consequently, the CET-Grid method was employed to identify these structural discontinuities in the potential field data.

This technique^20^ highlights areas with great structural complexity that could indicate prospective mineralization locations. This approach involves:

Texture highlighting emphasizes areas of complex patterns linked to discontinuities in gravity images by applying the Standard Deviation (SD), calculated using the following equation:

3 $$\sigma =\sqrt{[ \frac{1}{\mathrm{N}}{\sum } {\left({x}_{i}+\upmu \right)}^{2}]}$$

where, (N) is the number of cells with a mean value of 1, and (xi) are the values of the cells.

(3) Phase Symmetry (PS) – used to enhance the Standard Deviation (SD) findings for identifying zones of lateral discontinuity.

(3) Structure detection– employs the Phase Symmetry (PS) results to transform these discontinuity zones into linear structural features.

CET structural analysis parameters were selected after testing multiple window sizes and texture scales to optimize the delineation of fault intersections and structurally complex zones. Intermediate analysis scales produced the most coherent structural continuity and minimized noise amplification.

### Depth estimation techniques

Several quantitative interpretation methods were applied to estimate the depth and geometry of magnetic and gravity source bodies.

### Radially averaged power spectrum analysis

Radially averaged power-spectrum analysis was applied to estimate the average depths of shallow and deep sources. The method is based on analyzing the logarithmic decay of the energy spectrum with increasing wave number. This technique provides approximate depths for ensembles of causative sources and helps distinguish deep regional structures from shallow anomalies.

**Fig. 5:**
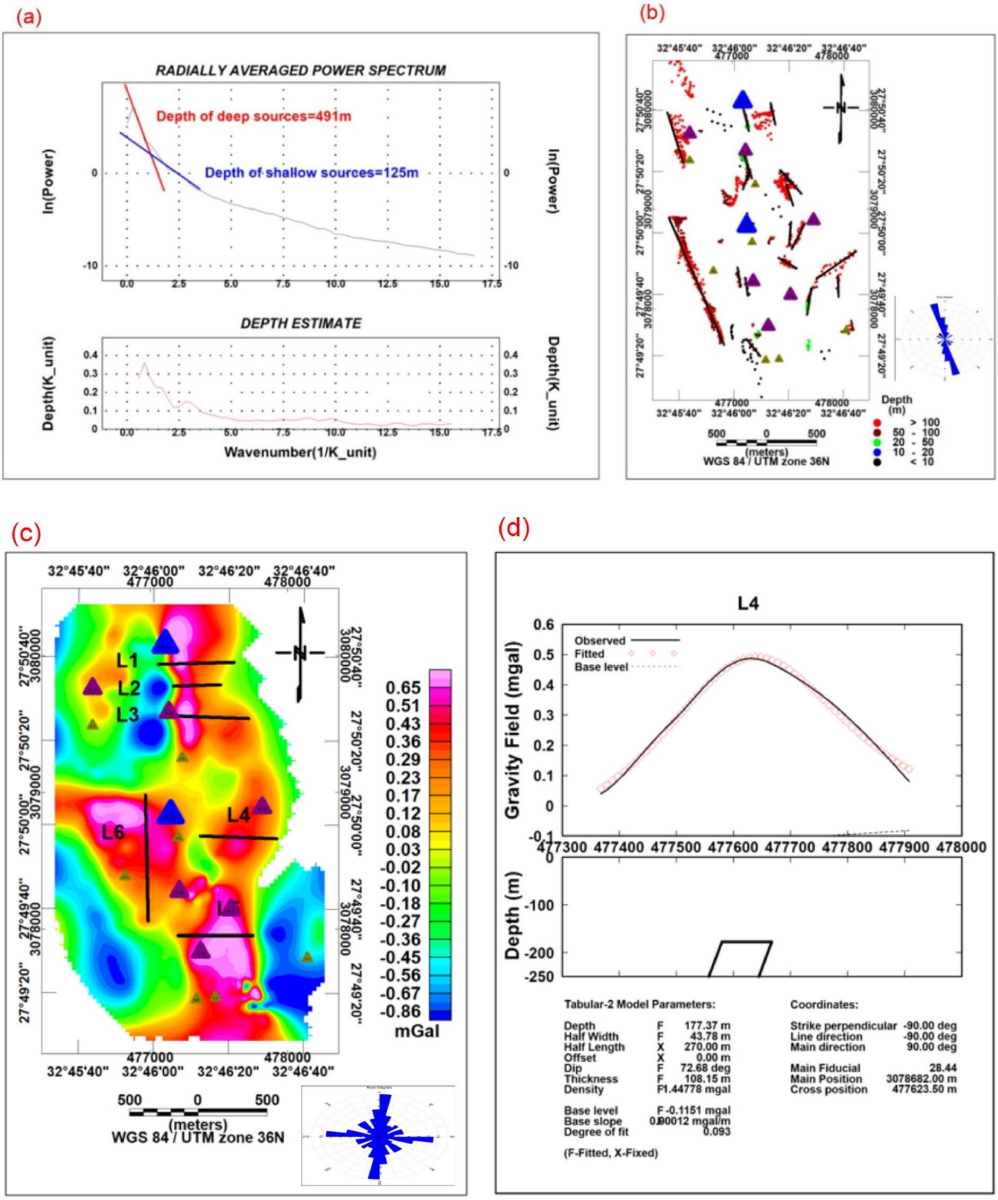
(**a**) Power spectrum curve for depth estimation, (**b**) Euler depth estimation map (Structural Index = 0), (**c**) Gravity residual anomaly map with model profile locations, (**d**) Example gravity model (Profile L4).

Depth to gravity sources was estimated using the radially averaged power spectrum method^18^ (Fig. 5a). The depth is calculated as:

4 $$z=-\frac{slope}{4\pi }$$

### Euler deconvolution for gravity data

Euler deconvolution was applied to estimate the locations and depths of subsurface source bodies using gravity field gradients. Structural indices corresponding to different geological models were tested to identify faults, contacts, and intrusive bodies. The method assists in locating subsurface structural discontinuities and estimating source depths without requiring prior geological constraints. Euler deconvolution was applied using a structural index (SI = 0) appropriate for contact^19^. The selection of processing parameters was based on the expected geological characteristics of the study area and the nature of the potential-field anomalies. For Euler deconvolution, a structural index (SI) of 0 was primarily adopted because the dominant gravity sources are interpreted as geological contacts and fault-controlled discontinuities. Previous studies demonstrated that SI = 0 is suitable for estimating depths associated with contact-type structures and sharp lithological boundaries within crystalline basement terranes.

5 $$\left(x-{x}_{0})\frac{\partial T}{\partial x}+(y-{y}_{0})\frac{\partial T}{\partial y}+(z-{z}_{0})\frac{\partial T}{\partial z}=N(B-T\right)$$

Clustered solutions (Fig. 5b) indicate depths ranging between less than 10 and more than 100 m.

Clustering statistics evaluated solution uncertainty; standard deviation within clusters was ± 10 m.

### Forward modeling

Two-dimensional forward modeling was conducted along selected profiles crossing major anomalies. Modeling was performed iteratively until calculated responses matched the observed gravity anomaly. The forward models were constrained using available geological information and structural interpretations. Model misfit was quantified using Root Mean Square Error (RMSE):

6 $$RMSE=\sqrt{\frac{1}{n}\sum ({T}_{obs}-{T}_{calc}{)}^{2}}$$

Final models achieved RMSE < 3% for gravity data (Table 1). Parameter sensitivity testing (± 10% variation) indicates depth uncertainty of ± 15 m and density uncertainty of ± 0.05 g/cm^3^.

**Table 1:** Results of gravity models.

Profile No	Longitude (X) UTM	Latitude (y) UTM	Depth (m)	Half Width (m)	Half Length (m)	Dip (deg)	Thickness (m)
L1	477,284.21	3,079,961.54	40	13.82	290	43.29	200.23
L2	477,316.467	3,079,811.01	70.68	32.27	175	54.15	159.62
L3	477,391.732	3,079,574.46	40.55	51.95	295	36.59	21.25
L4	477,628.281	3,078,682.02	177.37	43.78	270	72.68	108.15
L5	477,445.494	3,077,972.37	136.7	155.06	260	26.97	30.67
L6	476,972.395	3,078,574.5	100.63	36.78	485	140.14	522.32

The forward gravity models were generated iteratively using GravModel software constrained by available geological observations^31^ (Fig. 5c). Because potential-field inversion is inherently non-unique, the resulting subsurface geometries should be considered plausible rather than unique solutions. Residual fitting between observed and calculated anomalies was minimized during model optimization; however, uncertainties remain due to assumptions regarding density contrast, magnetic susceptibility, and body geometry.

### Mineralogical and geochemical analyses

#### ASD spectral interpretation

The ASD spectral signatures were interpreted using diagnostic absorption features to identify hydrothermal alteration minerals^14^. Spectral matching techniques were employed to compare field spectra with reference mineral libraries.

**Fig. 6:**
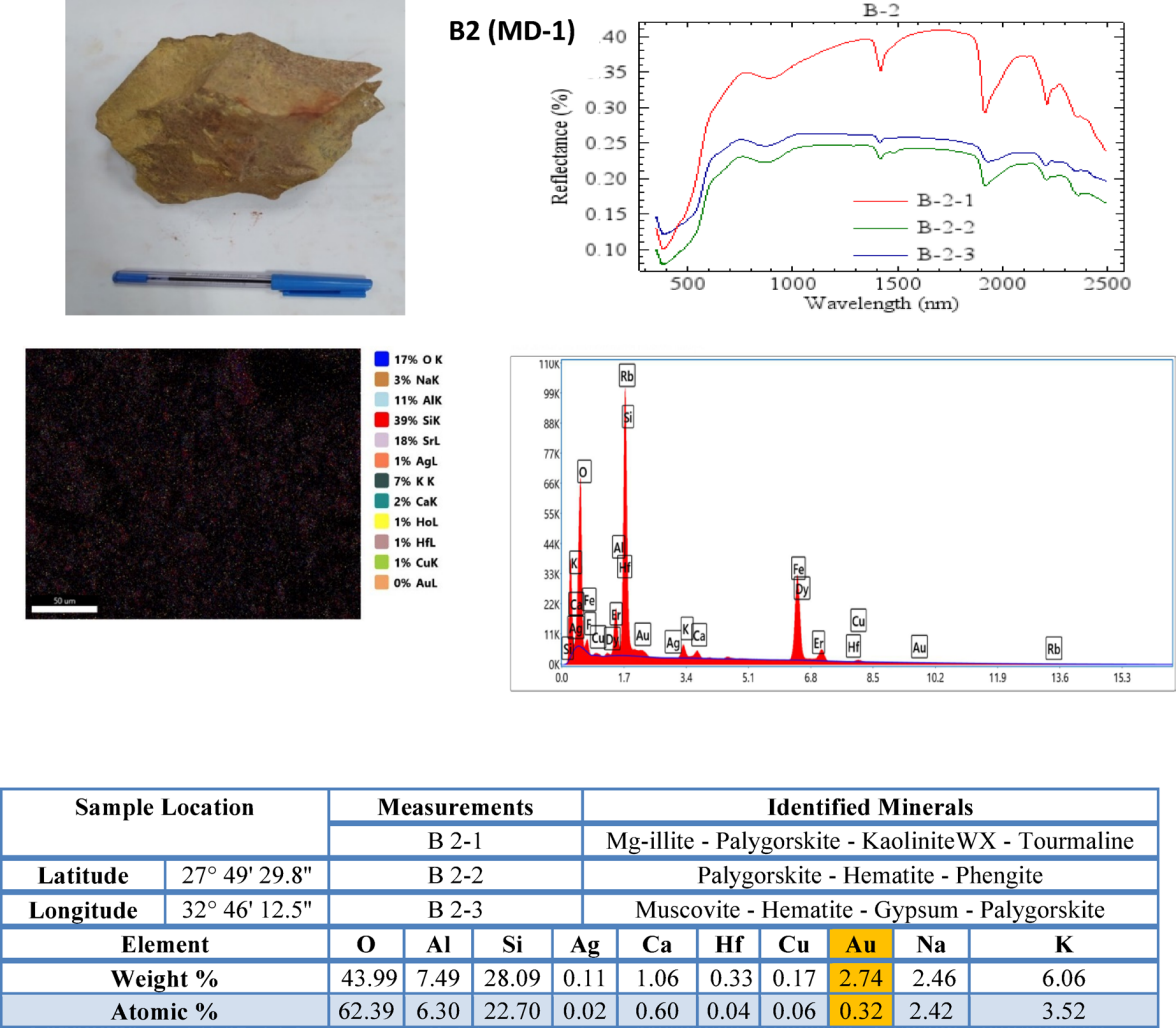
SEM spectra, SEM image, and summed spectrum of rock samples from the metagabbro–diorite complex.

**Fig. 7:**
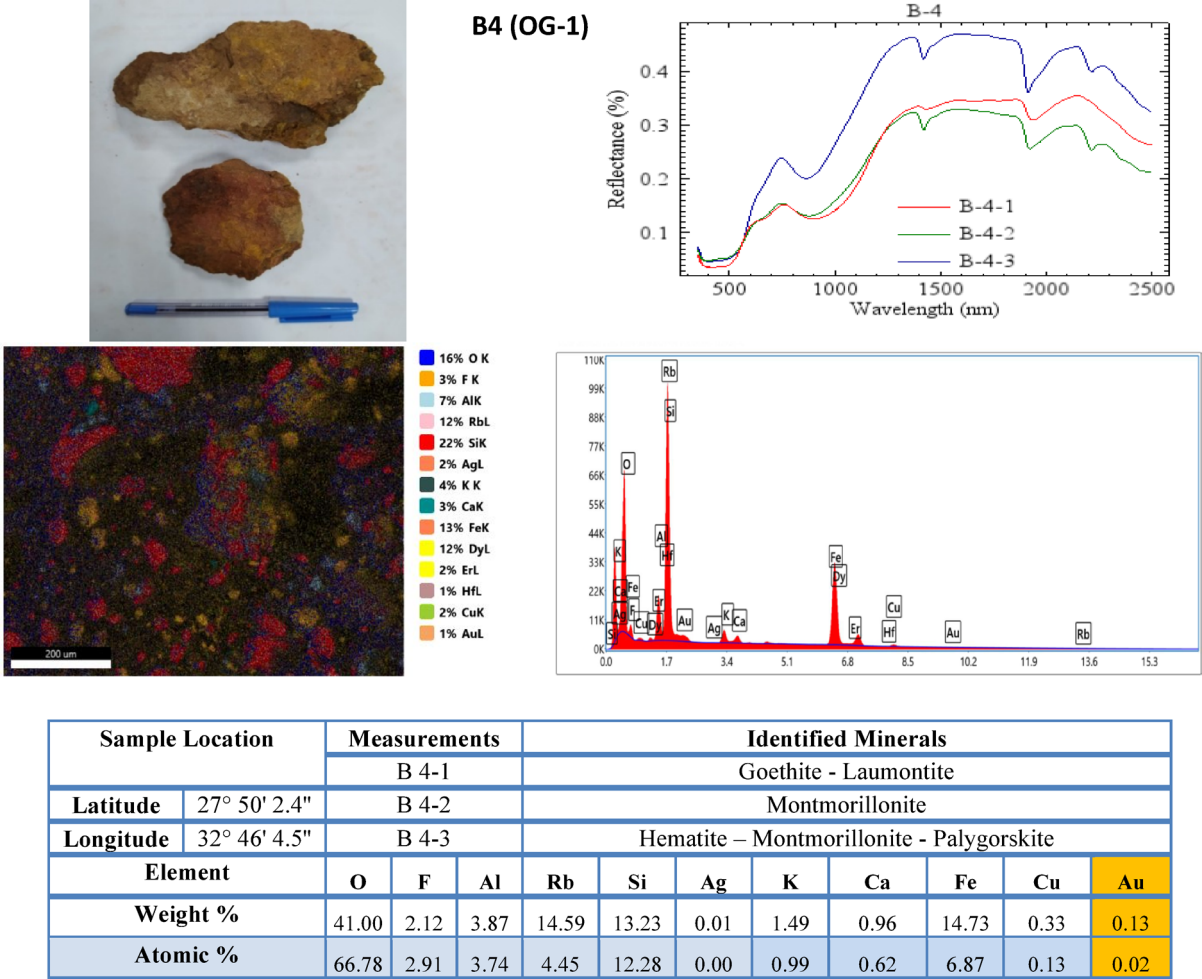
SEM spectra, SEM image, and summed spectrum of rock samples from older granitoids.

Minerals identified (Figs. 6 and 7) and Table 4) include: Hematite, Goethite, Illite, Kaolinite, Chlorite. Spectral matching accuracy using Spectral Angle Mapper (SAM) yielded classification confidence > 90%.

Band depth was calculated as:

7 $$BD=1-\frac{{R}_{c}}{{R}_{continuum}}$$

#### SEM–EDX analysis

SEM imaging was used to examine mineral textures, alteration features, and ore mineral associations (Figs. 6 and 7). ZAF correction was applied for quantitative analysis (Tables 2 and 3). EDX elemental analyses were conducted to determine the concentrations of major and trace elements associated with mineralization, including Au, Cu, Ag, Fe, and S. Integrating mineralogical and geochemical analyses provided constraints on hydrothermal alteration processes.

**Table 2:** Major element composition (Weight % and Atomic %) of analyzed samples.

Sample	Element	Fe	K	Si	O	Al	C	Ca	Mg	Na	Lithology
B2 (MD-1)	Weight %	-	6.06	28.09	43.99	7.49	-	1.06	-	2.46	Metagabbro-Diorite
	Atomic %	-	3.52	22.7	62.39	6.3	-	0.6	-	2.42	
B3 (MD-2)	Weight %	3.17	2.92	12.52	40.09	8.51	11.78	1.98	1.64	-	Metagabbro-Diorite
	Atomic %	1.23	1.62	9.67	54.33	6.84	21.27	1.07	1.46		
B7 (MD-3)	Weight %	-	2.36	26.85	39.03	7.38	8.2	-	-	-	Metagabbro-Diorite
	Atomic %	-	1.34	21.14	53.95	6.05	15.1	-	-	-	
B8 (MD-4)	Weight %	0.31	-	1.01	34.19	0.98	30.24	26.97	-	-	Metagabbro-Diorite
	Atomic %	0.1	-	0.66	39.18	0.66	46.16	12.34	-	-	
B9 (MD-5)	Weight %	5.6	3.72	31.66	36.64	9.42	-	0.67	-	-	Metagabbro-Diorite
	Atomic %	2.47	2.34	27.73	56.33	8.59	-	0.41	-	-	
B10 (MD-6)	Weight %	-	-	35.22	49.63	1.91	-	-	-	-	Metagabbro-Diorite
	Atomic %	-	-	26.22	64.86	1.48	-	-	-	-	
B13 (MD-7)	Weight %	-	-	40.02	53.79	-	-	1.13	-	1.16	Metagabbro-Diorite
	Atomic %	-	-	28.88	68.16	-	-	0.57	-	1.02	
B14 (MD-8)	Weight %	-	-	18.86	22.8	11.23	-	-	5.27	-	Metagabbro-Diorite
	Atomic %	-	-	21.52	45.68	13.34	-	-	6.94	-	
B16 (MD-9)	Weight %	9.06	0.34	8.11	30.53	1.59	34.17	0.41	-	-	Metagabbro-Diorite
	Atomic %	3.01	0.16	5.36	35.44	1.09	52.82	0.19	-	-	
B17 (MD-10)	Weight %	-	-	29.22	53.72		12.78	-	-	-	Metagabbro-Diorite
	Atomic %	-	-	18.94	61.15		19.37	-	-	-	
B18 (MD-11)	Weight %	-	-	21.71	41.27	0.64	27.08	-	-	-	Metagabbro-Diorite
	Atomic %	-	-	13.54	45.18	0.41	39.49	-	-	-	
B19 (MD-12)	Weight %	7.3	0.64	1.18	-	-	56.91	5.64	-	-	Metagabbro-Diorite
	Atomic %	2.42	0.31	0.78	-	-	87.82	2.61	-	-	
B20 (MD-13)	Weight %	-	-	32.32	45.5	-	18.41	-	-	-	Metagabbro-Diorite
	Atomic %	-	-	20.72	51.2	-	27.6	-	-	-	
B21 (MD-14)	Weight %	4.93	-	34.94	35.52	-	15.75	-	-	-	Metagabbro-Diorite
	Atomic %	1.8	-	25.29	45.13	-	26.66	-	-	-	
B4 (OG-1)	Weight %	14.73	1.49	13.23	41	3.74	-	0.96	-	-	Older-Granitoid
	Atomic %	6.87	0.99	12.28	66.78	3.74	-	0.62	-	-

**Table 3:** Trace element composition (Weight % and Atomic %) of analyzed samples.

Sample	Element	Au	Ag	Cu	Rb	F	Hf	Dy	Er	Ti	Lithology
B2 (MD-1)	Weight %	2.74	0.11	0.17	-	-	0.33	-	-	-	Metagabbro-Diorite
	Atomic %	0.32	0.02	0.06	-	-	0.04	-	-	-	
B3 (MD-2)	Weight %	3.72	0.08	1.49	-	-	-	6.79	0.49	-	Metagabbro-Diorite
	Atomic %	0.41	0.02	0.51			-	0.91	0.06	-	
B7 (MD-3)	Weight %	1.62	0.08	1.27	-	-	0.39	12.8	-	-	Metagabbro-Diorite
	Atomic %	0.18	0.02	0.44	-	-	0.05	1.74	-	-	
B8 (MD-4)	Weight %	0.36	0	0.08	-	-	-	-	-	-	Metagabbro-Diorite
	Atomic %	0.03	0	0.02	-	-	-	-	-		
B9 (MD-5)	Weight %	1.08	0.06	0.47	-	-	0.08	10	-	0.52	Metagabbro-Diorite
	Atomic %	0.13	0.01	0.18	-	-	0.01	1.53	-	0.27	
B10 (MD-6)	Weight %	5.69	0.22	-	-	-	1.3	-	-	-	Metagabbro-Diorite
	Atomic %	0.6	0.04	-	-	-	0.15	-	-	-	
B13 (MD-7)	Weight %	0.69	0.04	0.21	-	-	0.28	-	-	-	Metagabbro-Diorite
	Atomic %	0.07	0.01	0.07	-	-	0.03	-	-	-	
B14 (MD-8)	Weight %	0.14	0	0.25	-	-	-	-	-	-	Metagabbro-Diorite
	Atomic %	0.02	0	0.13	-	-	-	-	-	-	
B16 (MD-9)	Weight %	2.08	0.07	0.43	-	-	-	12.2	-	-	Metagabbro-Diorite
	Atomic %	0.2	0.01	0.13	-	-	-	1.4	-	-	
B17 (MD-10)	Weight %	2.03	0.22	0.56	-	-	1.47	-	-	-	Metagabbro-Diorite
	Atomic %	0.19	0.04	0.16	-	-	0.015	-	-	-	
B18 (MD-11)	Weight %	0.92	0.04	0.06	-	-	3.86	-	-	-	Metagabbro-Diorite
	Atomic %	0.08	0.01	0.02	-	-	0.38	-	-	-	
B19 (MD-12)	Weight %	0.12	-	0.04	19.68	-	-	-	0.28	-	Metagabbro-Diorite
	Atomic %	0.01	-	0.01	4.27	-	-	-	0.03	-	
B20 (MD-13)	Weight %	1.95	0.17	0.6	-	-	1.03	-	-	-	Metagabbro-Diorite
	Atomic %	0.18	0.03	0.17	-	-	0.11	-	-	-	
B21 (MD-14)	Weight %	0.75	0.11	0.16	-	-	0.17	7.66	-	-	Metagabbro-Diorite
	Atomic %	0.08	0.02	0.05	-	-	0.02	0.96	-	-	
B4 (OG-1)	Weight %	0.13	0.01	0.33	14.59	2.12	-	-	-	-	Older-Granitoid
	Atomic %	0.02	0	0.13	4.45	2.91	-	-	-	-	

## Results

The Um Balad area is situated in the North Eastern Desert of Egypt, within the tectonically complex Eastern Desert. The location map and geological setting (Fig. 1) show that the study area is mainly composed of metagabbro–diorite complexes and older granitoids dissected by major shear zones and structurally controlled gold occurrences. The geological relationships indicate strong structural control on lithological contacts and mineralized zones.

The total magnetic intensity (TMI) map (Fig. 2a) exhibits pronounced magnetic variations across the study area. Magnetic highs are predominantly concentrated in the central and northeastern sectors, whereas magnetic lows dominate the southwestern parts. The anomaly trends are mainly oriented NW–SE with subordinate NE–SW directions, reflecting the regional tectonic grain. The observed amplitude contrasts indicate significant differences in magnetic susceptibility. Magnetic lows are probably associated with altered, weathered, or felsic lithologies, while magnetic highs are thought to be shallow mafic intrusions or magnetite-rich metagabbro–diorite units. After applying reduction to the pole (RTP) (Fig. 2b), the anomalies become symmetrically positioned over their causative sources, allowing more accurate delineation of magnetic bodies. The RTP map clearly defines compact magnetic highs that are interpreted as structurally controlled intrusions. Spectral analysis of the magnetic and gravity data (Figs. 2c and 5a) reveals two principal depth levels. The shallow sources range from approximately 94 to 125 m, corresponding to near-surface intrusions and altered zones, while deeper sources extend from about 187 to 400 m, representing deeper basement structures.

The magnetic residual map (Fig. 3a) enhances shallow anomalies and clearly outlines fault-controlled magnetic highs. In contrast, the regional magnetic component (Fig. 3b) reflects deeper crustal features. By applying the CET-Porphyry method to the magnetic map (Fig. 3c), a map of the research area’s porphyry enhancement (Fig. 2b) is generated. The weak porphyry intrusions in the region are depicted on this map. These features are primarily concentrated in the central area, with some appearing at sporadic sites elsewhere. The CET porphyry feature extraction and tilt derivative maps (Fig. 3c and d) emphasize structural contacts and fault systems, particularly along NW–SE and NE–SW orientations. These structural trends spatially coincide with known mineralized zones, indicating a strong relationship between magnetic lineaments and hydrothermal activity.

The Bouguer gravity anomaly map (Fig. 4a) shows moderate to high gravity values in the central and northeastern parts of the area, which correlate well with magnetic highs. These gravity highs indicate the presence of dense subsurface bodies, most likely mafic and intermediate intrusive rocks. Lower gravity anomalies in the western and southern sectors suggest less-dense lithologies or structurally downfaulted basement blocks. Structural enhancement techniques, including CET structural analysis (Fig. 4b), tilt derivative (Fig. 4c), and first vertical derivative (Fig. 4d), clearly delineate major fault systems and lithological boundaries.

The Circular Feature Transform (CET) is an image-processing technique for identifying circular or arcuate structural features in geophysical datasets. The method enhances localized circular patterns by analyzing pixel orientation and curvature distributions within moving windows.

The main structural trends identified by the CET structural map are NNW, N-S, E-W, and NW (Fig. 4b). The rose diagrams of the zero-contours from the TDR and FVD maps indicate that the predominant structural trends are NNW–SSE and NNE–SSW, respectively (Figs. 4c and d). The radial average power spectrum of gravity data indicated that the depths of the deep and shallow sources are 491 m and 125 m, respectively. These results are further supported by Euler depth estimation (Fig. 5b), which shows clustered solutions concentrated along important structural corridors and varying in depth from roughly less than 10 to more than 100 m. The gravity residual map (Fig. 5c) highlights shallow density contrasts, while forward modeling along profile L4 (Fig. 5d) indicates tabular subsurface bodies with significant density contrast relative to surrounding rocks.

Table 1 summarizes the gravity-modeled properties, confirming the presence of high-density bodies corresponding to metagabbro–diorite intrusions, with depths to the top ranging from 40 m to 177.37 m, and an average of 94.3 m. These results indicate that the mineralized sources beneath the study area are relatively shallow. The depth framework obtained using the Euler technique (Fig. 5b) in the Um Balad area aligns with the Gravmode inversion results (Fig. 5c and Table 1) for the high-pass gravity anomalies, which show an average depth of 94.3 m. Additionally, it confirms the power-spectrum-based depth estimates (Fig. 5a) for the buried gravity bodies, indicating that the mineralized sources are located at relatively shallow depths, with an average depth of 125 m. Furthermore, the structural trend systems derived using high-pass gravity filters and FVD gravity techniques (Figs. 4b and5c) are supported by the trends of solutions obtained by the Euler technique (Fig. 5b), which indicate NW–SE, NNW-SSE, and NE-SW as the primary bearings of gravity sources.

The geochemical characteristics of the metagabbro–diorite samples exhibit considerable variations in elemental composition based on SEM–EDS analyses (Figs. 6 and 7). The analyzed samples are generally enriched in Si, O, Al, Fe, K, Ca, and C, reflecting extensive hydrothermal alteration and silicification processes. Copper concentrations range from 0.04 to 1.49 wt.%, whereas Au and Ag contents range from 0.12 to 5.69 wt.% and 0.00 to 0.22 wt.%, respectively. The elevated Si and O contents indicate the predominance of silica-rich phases, while Fe-bearing phases are represented by hematite, goethite, ferrihydrite, and jarosite identified by mineralogical analyses.

Fourteen representative hand specimens (MD-1 to MD-14) were collected from the alteration zones within the metagabbro–diorite rocks and investigated using SEM–EDS analysis. Samples MD-2 and MD-12, collected from the host metagabbro–diorite, contain Au concentrations of 3.72 wt.% and 0.12 wt.%, respectively. Among all analyzed samples, MD-6 exhibits an anomalously high Au concentration reaching 5.69 wt.% (Table 3). Mineralogical investigations further reveal the presence of hydrothermal clay minerals (illite, kaolinite, montmorillonite, and palygorskite), zeolite minerals (chabazite, laumontite, and heulandite), and oxidation-related minerals such as hematite and goethite, indicating multistage hydrothermal alteration under oxidizing conditions (Table 4).

**Table 4:** Mineral contents.

Sample	Measurements	Identified Minerals
B2 (MD-1)	1	Mg-illite—Palygorskite—KaoliniteWX—Tourmaline
	2	Phengite—Palygorskite –Hematite
	3	Muscovite—Hematite—Gypsum—Palygorskite
B3 (MD-2)	1	FeMgChlorite—Phillipsite-Ca—Gypsum—FeChlorite—Hydrozincite
	2	Goethite – Phengite—Chabazite
	3	Mg-illite—FeChlorite—Goethite—Hydrozincite- Heulandite
B7 (MD-3)	1	Phengite—Chabazite
	2	Phengite—Chabazite
	3	Vermiculite—K-illite
B8 (MD-4)	1	Rhodochrosite—Calcite
	2	Strontianite—Mg-illite
B9 (MD-5)	1	Goethite—Hydrozincite—Mg-illite
	2	Ferrihydrite—Muscovite—Mg-illite
	3	Muscovite—Mg-illite—Goethite
B10 (MD-6)	1	Laumontite
	2	Hematite—Laumontite
	3	Dioptase—Laumontite
B13 (MD-7)	1	Hydrozincite—FeChlorite—Gmelinite-Na
	2	K-illite—Goethite—FeMgChlorite—Heulandite—Dickite—Gypsum
	3	Montmorillonite—Ferrihydrite—FeChlorite—KaoliniteWX
B14 (MD-8)	1	Hematite
	2	Hematite
	3	Hematite
B16 (MD-9)	1	Goethite, Natrojarosite, Palygorskite
	2	Chabazite
	3	Goethite, Jarosite
B17 (MD-10)	1	Hematite, Laumontite
	2	Chabazite, Phengite
	3	Hematite, Laumontite
	4	Chabazite
	5	Hematite, Chabazite, Epidote
B18 (MD-11)	1	Chabazite
	2	Hematite, Chabazite, Dioptase, Gypsum
	3	Hematite, Chabazite
B19 (MD-12)	1	Ferrihydrite, Palygorskite, Clinozoisite, KaolinitePX, Hematite
	2	Ferrihydrite, Palygorskite, Tourmaline, FeChlorite, Goethite
	3	Montmorillonite, KaoliniteWX
B20 (MD-13)	1	Chabazite
	2	Chabazite, Dioptase, Gypsum
	3	Chabazite, Gypsum, Dioptase, Dolomite
B21 (MD-14)	1	Gmelinite-Na, FeMgChlorite, Azurite
	2	Mg-illite, KaoliniteWX, Palygorskite, FeChlorite
B4 (OG-1)	1	Laumontite—Goethite
	2	Montmorillonite
	3	Palygorskite – Montmorillonite- Hematite

Regarding the older granitoids, one representative sample collected from the alteration zone was analyzed Fig. 7 and (Table 2). The sample is characterized by Au and Ag concentrations of 0.13 wt.% and 0.01 wt.%, respectively, in addition to relatively elevated Fe (14.73 wt.%), Na (14.59 wt.%) (Table 2), and F (2.12 wt.%) contents (Table 3). Mineralogically, the sample contains laumontite, montmorillonite, palygorskite, goethite, and hematite, suggesting hydrothermal alteration accompanied by zeolitization and oxidation processes (Table 4).

The results of the mineralogical analysis based on SEM spectra and images are shown in Figs. 6 and 7, and (Table 4). The results show that both the metagabbro–diorite complex and the older granitoids have undergone significant hydrothermal alteration. The identified minerals are hematite, goethite, ferrihydrite, Mg-illite.

The presence of iron oxide minerals, such as hematite and goethite, indicates oxidation reactions resulting from hydrothermal and possibly supergene alteration. Clay minerals such as illite, kaolinite, and montmorillonite indicate argillic alteration zones that are commonly associated with hydrothermal fluid-rock interaction. Zeolite minerals such as laumontite and chabazite indicate hydrothermal fluids under relatively low temperatures, whereas carbonate minerals such as rhodochrosite and calcite indicate hydrothermal fluids involving carbonate alteration.

The geochemical analysis of samples B2 to B21 and B4 reveals considerable variations in elemental composition. Gold concentrations range from 0.12 to 5.69 wt.%, with the highest Au concentration recorded in sample B10 (MD-6). Relatively elevated Au contents are also observed in samples B3, B2, B16 and B17. The analyzed samples contain several associated elements, including Ag, Cu, Fe, Si, Al, K, Ca, Dy, and Hf (Table 2).

The elevated Fe (Table 2) content is attributed to the occurrence of iron oxide and hydroxide minerals such as hematite and goethite identified by mineralogical analyses. The coexistence of K, Al, and clay minerals (Table 2) indicates the development of potassic, argillic, and phyllic hydrothermal alteration. The presence of Cu and Ag (Table 3) suggests the influence of polymetallic hydrothermal fluids associated with the mineralization processes. In addition, the occurrence of Dy and Hf (Table 3) may reflect the presence of accessory heavy minerals possibly inherited from magmatic processes.

The integrated analysis of Au concentrations with Bouguer gravity and RTP magnetic data shows a clear spatial association between gold mineralization and geophysical anomalies. Higher gold values (MD-6, MD-2, MD-1) correspond to moderate–high magnetic responses and locally positive gravity anomalies, suggesting structurally controlled zones enriched in hydrothermal alteration and denser lithologies such as mafic intrusions or shear zones. In contrast, low Au samples are linked to weak or negative geophysical signatures Table 5.

**Table 5:** Sample coordinates and associated geophysical values.

St	Long (Degree)	Lat (Degree)	AU Weight %	Bouguer	RTP
B2 (MD-1)	32.770	27.825	2.74	-0.18	42,737
B3 (MD-2)	32.768	27.834	3.72	0.66	42,819
B7 (MD-3)	32.762	27.842	1.62	0.11	42,681
B8 (MD-4)	32.769	27.838	0.36	1.59	42,870
B9 (MD-5)	32.768	27.841	1.08	1.79	42,867
B10 (MD-6)	32.767	27.845	5.69	3.28	42,830
B13 (MD-7)	32.765	27.830	0.69	-0.73	42,796
B14 (MD-8)	32.778	27.825	0.14	-0.94	42,643
B16 (MD-9)	32.772	27.828	2.08	0.37	42,700
B17 (MD-10)	32.775	27.835	2.03	2.39	42,812
B18 (MD-11)	32.762	27.84	0.92	-0.61	42,625
B19 (MD-12)	32.77	27.822	0.12	-1.28	42,680
B20 (MD-13)	32.769	27.829	1.95	-0.13	42,811
B21 (MD-14)	32.771	27.822	0.75	-0.88	42,675
B4 (OG-1)	32.768	27.833	0.13	0.51	42,814

From a spatial viewpoint, areas of the highest Au concentration are associated with structural controls and significant magnetic and gravity anomalies. The above findings indicate a genetic relationship with structural deformation, magmatic activity, and hydrothermal mineralization. In conclusion, after analyzing magnetic, gravity, mineralogical, and geochemical data, the Um Balad area is characterized by structural controls related to mafic intrusive activity and hydrothermal alteration. Areas related to the N-S and NW–SE faults are primarily favorable for mineralization, whereas those related to NE-SW structures are favorable for permeability and gold mineralization within the metagabbro–diorite complex of the Um Balad area.

### Spatial integration of geophysical and geochemical data

To evaluate the relationship between hydrothermal mineralization and subsurface structural features, sample locations were superimposed on the RTP magnetic and Bouguer gravity anomaly maps (Figs. 2a and 4b). Gold-enriched samples are preferentially concentrated along structurally complex zones characterized by magnetic discontinuities, gravity gradients, and intersecting NW–SE and NE–SW fault systems.

Sample B10 (MD-6), which yielded the highest Au concentration, is spatially associated with a moderate magnetic gradient zone and a localized gravity transition interpreted as a structurally controlled hydrothermal corridor (Table 5). The coincidence between hydrothermal alteration minerals, elevated Au concentrations, and geophysical discontinuities suggests that fault-controlled permeability played an important role in hydrothermal fluid migration and mineral deposition.

However, the relationship between geophysical anomalies and mineralization is not necessarily unique, as lithological contacts and non-mineralized intrusive bodies can also contribute to the observed anomalies. Therefore, the integrated interpretation identifies structurally favorable exploration targets rather than definitive ore bodies.

### Limitations of the study

Several limitations should be considered when interpreting the present results. The airborne magnetic dataset is constrained by the original survey resolution and flight-line spacing, which may limit the detection of small-scale structural features. Furthermore, potential-field interpretation methods are inherently non-unique and depend on assumptions regarding source geometry, density contrast, and magnetic susceptibility. The mineralogical and geochemical interpretations are based on a limited number of representative samples and localized SEM–EDX spot analyses rather than systematic bulk-rock assays. In addition, no drilling or trenching data were available to validate the interpreted subsurface anomalies and modeled structures directly.

Consequently, the identified targets should be regarded as structurally favorable exploration zones requiring additional field verification, detailed geochemical sampling, and subsurface investigation.

## Discussion

The integrated geophysical, mineralogical, and geochemical results from the Um Balad area provide strong evidence that gold mineralization is structurally controlled and genetically related to magmatic–hydrothermal processes within the Neoproterozoic basement of the Eastern Desert, Egypt. The regional geological framework (Fig. 1) indicates that the study area lies within a tectonically deformed segment of the Arabian–Nubian Shield, where major shear zones and intrusive complexes are closely associated with gold occurrences. The magnetic results (Figs. 2 and 3) reveal dominant NW–SE and subordinate NE–SW structural trends. These orientations coincide with the principal deformation phases documented in the Eastern Desert and reflect regional-scale shear systems. The clustering of high-pass and tilt derivative maps (Fig. 3a and d) along these structural corridors suggests that magnetic bodies are emplaced or modified along fault zones. Such structural control is characteristic of orogenic gold systems, where deformation zones act as conduits for hydrothermal fluids. The strong magnetic highs associated with metagabbro–diorite bodies imply the presence of magnetite-rich intrusions that may have provided thermal energy and fluid sources for mineralization.

Gravity data further support this interpretation. The Bouguer anomaly map and its derivatives (Fig. 4) show dense subsurface bodies spatially correlated with magnetic highs, indicating mafic intrusive complexes. Forward modeling results (Fig. 5d and Table 1) confirm the presence of tabular, high-density bodies consistent with intrusive emplacement along fault systems. The coincidence of high-density and high-magnetic anomalies suggests that magmatic processes played a fundamental role in the area’s structural evolution. Similar relationships between intrusions and mineralization have been reported in comparable gold systems of the Eastern Desert, where magmatic-hydrothermal interaction enhances metal concentration.

Mineralogical data (Figs. 6 and 7) and Table 4 demonstrate widespread hydrothermal alteration affecting both the metagabbro–diorite complex and older granitoids. The abundance of iron oxides (hematite, goethite, ferrihydrite) indicates oxidation and supergene modification, whereas clay minerals such as illite, kaolinite, montmorillonite, and phengite reflect argillic and phyllic alteration. The presence of zeolites (laumontite, chabazite) suggests low-temperature hydrothermal conditions during late alteration stages. Carbonate minerals such as rhodochrosite and calcite point to carbonate alteration, which is commonly associated with fluid–rock interaction in structurally controlled gold systems. This mineral assemblage collectively indicates a multi-stage hydrothermal evolution involving potassic, phyllic, and argillic alteration phases.

Geochemical data reinforce the proposed hydrothermal mineralization model (Figs. 6 and 7; Table 2). Elevated gold values, particularly in samples B10 (MD-6), B3 (MD-2), and B2 (MD-1), are spatially associated with iron oxides and hydrothermal clay alteration zones. The enrichment of Fe, Cu, and Ag alongside Au suggests polymetallic hydrothermal activity. Potassium enrichment corresponds with illite- and phengite-bearing alteration assemblages, indicating potassic metasomatism. The detection of trace elements such as Dy and Hf (Table 3) may indicate the contribution of accessory magmatic-related minerals, suggesting that the mineralizing fluids were at least partially influenced by intrusive sources. Furthermore, the spatial coincidence between geochemical anomalies and magnetic and gravity highs suggests a structural and magmatic control on the mineralization processes.

The integration of all datasets indicates that the Um Balad region’s mineralization is localized at the intersection of major NW–SE shear zones with secondary NE–SW structures. These intersections enhance permeability and fluid focusing, creating favourable sites for gold deposition. The presence of shallow magnetic and gravity sources, as indicated by spectral analysis and modeling (Figs. 2 and 5), suggests that mineralization is relatively shallow and structurally confined.

Overall, the geophysical signatures, alteration mineral assemblages, and geochemical enrichment patterns collectively support a magmatic–hydrothermal model for gold mineralization in the Um Balad area. Mafic-to-intermediate intrusions provided heat and possibly metals, while regional shear systems served as conduits for fluid migration and deposition. The strong correlation among structural trends, high-density/magnetic bodies, and alteration zones underscores the exploration significance of integrated geophysical and geochemical approaches for delineating prospective gold targets in the Eastern Desert.

## Conclusion

This study integrated airborne magnetic data, ground gravity measurements, ASD spectral analyses, and SEM–EDX investigations to examine the structural framework and hydrothermal alteration associated with gold mineralization in the Um Balad area, northern Eastern Desert, Egypt. The combined interpretation improved the characterization of lithological boundaries, structural discontinuities, and alteration zones within the study area. Magnetic and gravity enhancement techniques revealed dominant NW–SE, NNW–SSE, and NE–SW structural trends that appear to control the distribution of hydrothermal alteration zones and quartz-vein systems. Depth estimation methods, including radially averaged power spectrum analysis, Euler deconvolution, and forward modeling, suggest the presence of both shallow and relatively deeper magnetic and density source bodies. However, these interpretations represent approximate subsurface geometries constrained by the resolution and assumptions of the applied geophysical methods.

The different depth estimation methods used in this study provide complementary rather than identical information. The radially averaged power spectrum analysis yielded average source depths of approximately 125 m and 491 m, representing statistical estimates of shallow and deeper regional source ensembles. In contrast, Euler deconvolution preferentially identified localized shallow discontinuities ranging from less than 10 m to more than 100 m depth, while forward modeling constrained intermediate-depth causative bodies between approximately 40 and 177 m. These differences are expected because each technique responds to different wavelength components, source geometries, and spatial scales.

Mineralogical investigations identified alteration assemblages dominated by hematite, goethite, chlorite, kaolinite, muscovite, illite, and ferrihydrite, indicating hydrothermal fluid–rock interaction within structurally deformed zones. SEM–EDX analyses revealed localized enrichments of Au, Cu, and Ag in selected samples, although their spatial continuity and economic significance require additional verification through detailed sampling and drilling. The spatial association between structural complexity, geophysical discontinuities, and hydrothermal alteration suggests that fault intersections and deformation corridors may have acted as pathways for hydrothermal fluid migration. Nevertheless, the direct relationship between geophysical anomalies and mineralization should be interpreted cautiously, as potential-field anomalies may also reflect lithological contrasts, variations in alteration intensity, or non-economic intrusive features.

Rather than identifying definitive ore bodies, the integrated workflow presented in this study provides a framework for delineating structurally favorable hydrothermal targets within crystalline basement terranes. The results demonstrate the value of combining geophysical, mineralogical, and geochemical datasets to reduce interpretational ambiguity and improve exploration targeting within the Arabian–Nubian Shield.

Future investigations should incorporate higher-resolution geophysical surveys, quantitative inversion approaches, hyperspectral remote sensing, isotopic analyses, and drilling data to better constrain subsurface geometry, fluid evolution, and mineralization processes in the Um Balad area and comparable Precambrian mineral provinces.

## Data Availability

The data that support the findings of this study are available from the corresponding author upon reasonable request.
